# Prospective validation of the “rhino conjunctivitis allergy-control-SCORE^©^” (RC-ACS^©^)

**DOI:** 10.1186/2045-7022-2-17

**Published:** 2012-09-19

**Authors:** Dietrich Häfner, Kristian Reich, Ina Zschocke, Annett Lotzin, Hanns Meyer, Jens Kettner, Annemie Narkus

**Affiliations:** 1 Medical Department, Allergopharma J. Ganzer KG, Hermann-Körner-Str. 52, Reinbek, 21465, Germany; 2 Dermatologikum Hamburg, Hamburg, Germany

**Keywords:** Symptom score, Medication score, Allergic disease, Rhino conjunctivitis, Symptom severity

## Abstract

**Background:**

Recently we reported the validation of the “Allergy-Control-SCORE^©^ (ACS)” which assesses symptom severity as well as medication use on three dimensions lung, nose and eyes. The aim of this study was to test the validity of the score for eyes and nose.

**Methods:**

One-hundred-twenty-one consenting subjects (age 19-65y), including 81 patients with allergic rhino-conjunctivitis (RC) and 40 healthy controls, participated in the study. Patients rated daily nasal and eye symptoms using a 4-point scale (none, mild, moderate, and severe) and their use of anti-symptomatic medication. Validation criteria were pollen counts in the course of the study period. Discrimination capacity was analyzed by comparing the rhino-conjunctivitis Allergy-Control-SCORE^©^ (RC-ACS^©^) values of allergic patients and healthy controls. Convergent reliability was assessed by correlating RC-ACS^©^ values with the global severity of allergy, the quality of life, and the allergy-related medical consultations. Retest reliability was assessed by the correlation of the repeated measured RC-ACS^©^ scores during each of two consecutive weeks.

**Results:**

Convergent reliability analysis indicated a significant correlation between RC-Allergy-Control-SCORE^©^ and global severity of allergy (*r* = 0.691; *p* < 0.0001), quality of life (*r* = 0.757; *p* < 0.0001) and allergy-related medical consultations (*r* = 0.329; *p* = 0.0019). RC-Allergy-Control-SCORE^©^ showed a good retest reliability (*r* = 0.813; *p* < 0.001) and discriminated extremely well between allergic patients and healthy controls (Median: 3.7 range: 0; 14.1 vs. Median: 0 range: 0; 2.9; p < 0.001), with a sensitivity of 93.8% and a specificity of 92.5% at a score value of 0.786.

**Conclusions:**

The RC-ACS^©^ can be considered as valid and reliable to assess the severity of rhino-conjunctivitis severity in clinical trials and observational studies.

## Background

Recently we reported the validation of the “Allergy-Control-SCORE^©^ (ACS)” which includes three categories: lung, nose and eyes [1]. Now we report the validation of the score for eyes and nose, only. With this new score we suggest an approach which covers a symptom score, and a medication score, for eyes and nose symptoms and use of symptomatic allergy medication to a combined symptom-medication score (SMS), the Rhino-Conjunctivitis Allergy-Control-Score (RC-ACS^©^). Such a SMS is recommended to measure the primary outcome of clinical trials on respiratory allergies [2], and the use is proposed by international regulatory agencies, e.g. European Medicines Agency (EMA) [3].

With this validation we also report the outcome of the validation of the Eye-Allergy-Control-Score (E-ACS^©^) and the Nose-Allergy-Control-Score (N-ACS^©^). The Allergy-Control-Score (ACS^©^). was used for several years in different clinical trials [4-6]. In these studies the score was used under the synonym, symptom medication score. The E-ACS^©^ and the N-ACS^©^ as well as the RC-ACS^©^ are parts of the Allergy-Control-SCORE^©^. The RC-ACS^©^ covers drugs used in clinical trials and observational studies.

The concept of validation of the RC-ACS^©^ is based on measuring 1) convergent reliability and 2) retest reliability in allergic patients as well as 3) discrimination capacity in healthy controls and patients with respiratory allergies. Therefore a prospective study was designed which included patients suffering from allergic rhinitis, conjunctivitis or both and healthy controls. The convergent reliability was assessed to determine the degree to which the scores of an instrument show a relationship to scores of similar instruments. For this purpose the correlation to the following two instruments and two clinically important disease related health economic measures was assessed: 1) Global Assessment of Severity of Allergy by use of a Rating Scale; 2) Quality of Life (RQLQ); 3) number of medical consultations due to the allergy within the last 12 months; 4) the number of non-productive days due to allergic rhino-conjunctivitis and/or asthma within the last 12 months. Retest reliability is the extent to which scores for patients who have not changed are the same for repeated measurement over time. The retest reliability was determined by correlating the SMS values of the first week with the values of the second week. The discrimination capacity reflects the degree to which the scores of an instrument can discriminate between different patient groups. Discrimination capacity was assessed by comparing the average SMS values of the allergic patients (first week) and the control group, respectively.

The validation of a Rhino-Conjunctivitis Allergy-Control-Score (RC-ACS^©^) is an important and topical issue in allergy clinical research. It is a relevant instrument to assess rhino-conjunctivitis severity in clinical trials and observational studies. With the validation a formal aspect for reliable use of such an instrument is fulfilled.

This Rhino-Conjunctivitis Allergy-Control-Score (RC-ACS^©^) introduces the concept of “control of disease (rhino-conjunctivitis)” as this will be the aim when using any therapeutic intervention. The presented score balances symptoms and use of medication and it also considers the influence of treatment on allergic symptoms. Therefore, for calculating the combined symptom medication score, each medication will be assessed e.g. according to the use, the specific effects and the way of administration.

## Methods

### Study population

Patients (age 19 to 65 years) were recruited from the outpatient clinic department of “Dermatologikum Hamburg”, Germany, between 21 June and 17 August 2008. Inclusion criteria were: 1) atopic sensitization (SPT positivity to at least one of the following allergens: grass, rye, mugwort pollen, house dust mites) (Allergopharma J. Ganzer KG, Reinbek, Germany); 2) current clinical manifestations of allergic rhino-conjunctivitis and/or asthma due to exposure to one of the four allergens listed above; 3) expected natural exposure to the relevant allergens during the study period. Controls were non-atopic volunteers with a negative history for IgE-mediated allergies. The following exclusion criteria were applied to patients and controls: 1) current use of systemic or nasal corticosteroids, inhaled corticosteroids (>400μg budesonide or >500μg beclomethasone dipropionate per day); 2) long-term prophylactic use of anti-allergic medication with constant dose; 3) current treatment with specific immunotherapy; 4) food allergy; 5) clinically-relevant rhinitis/rhino-conjunctival or respiratory symptoms related to other unidentified causes; 6) vasomotor, drug-induced or other kinds of non-allergic rhinitis/rhino-conjunctivitis; 7) febrile infections or inflammation of the respiratory tract; 8) irreversible secondary alterations of the upper and lower airways (e.g. emphysema, bronchiectasis etc.). The study protocol was discussed with the local Ethics Committee before it was commenced. The committee advised that formal approval was not required, because the study was observational and no changes in treatment were involved. However, written informed consent was received from all patients involved before they were included into the study.

### Study design

The study was designed as prospective, observational and controlled study. Patients and healthy controls completed a questionnaire on demographic and clinical parameters at recruitment. Scores of individual symptoms and individual medications were documented daily during the pollen season over a period of 2 weeks in patients and 1 week in healthy controls, respectively. On each day, patients and controls were also asked to complete a self-administered questionnaire for a “Global Assessment of Severity of Allergy”. This was performed through a Visual Rating Scale (rating scale ranging from 1 = no symptoms at all to 10 = very severe symptoms), which is similar to the one described by Bousquet and colleagues [7]. At the end of the first week, patients completed a validated questionnaire to rate their quality of life (RQLQ^©^) [8]. Participants started at different times during the season, so that both study groups included subjects that were exposed to high or low pollen counts. In addition, patients were asked how many days they were incapable of working due to their allergic rhino-conjunctivitis and/or asthma within the last 12 months (number of non-productive day).

### RC-ACS^©^

#### Symptom score

The characteristics of the Rhino-Conjunctivitis Allergy-Control-SCORE^©^ as listed in Table 1 were elaborated according to the GA(2)LEN taskforce Guidelines [9]. The symptom score is recorded using diaries in which subjects documented daily the severity of various allergy symptoms scaled according to the EMA guidelines [3] on a scale ranging from 0 to 3: 0 = absent (no sign/symptom evident); 1 = mild (sign/symptom clearly present, but minimal awareness; easily tolerated); 2 = moderate (definite awareness of sign/symptom that is bothersome, but tolerable); 3 = severe (sign/symptom that is hard to tolerate; causes interference with activities of daily living and/or sleeping). For each day, the sum of the values of the seven allergy symptoms is calculated. These include ocular (itching, tear flow, redness), and nasal (sneezing, itching, running, blockage) symptoms.

**Table 1:** **Characteristics of the Rhino-conjunctivitis allergy control score (RC-ACS)**^**©**^**, according to the GA2LEN recommendations **[8]

	
Acronym	Rhino-Conjunctivitis Allergy Control Score (RC-ACS^©^)
Author	Kettner J., Narkus A., Häfner D.
Target	To objectively monitor severity of allergic rhinitis and allergic conjunctivitis
Population	Adult and adolescent patients with allergic rhinitis/conjunctivitis
Administration	Patient diaries
Original language	English
Existing translations	German, Polish and others
Number of items	7 symptoms and 745 drugs
Tool dimensions	see methods
Scaling of items	Score points
Scoring of items	0-42 (global)
List of items	see methods
Minimal important difference	To be determined
Shortened versions	none
Performed trials	Validation and use in different clinical trials
Copyright	Allergopharma Joachim Ganzer KG
Contact information	Häfner D. Medical Department Allergopharma J. Ganzer KG, Hermann-Körner-Str. 52 21465 Reinbek e-mail: dietrich.haefner@allergopharma.de

#### Medication score

Patients also have to document the allergy medication needed. All allergy medications for treating related symptoms are scored for each patient and each available day. Categories of medication taken into consideration include nasal and ocular anti-histamines and glucocorticoids, nasal decongestants, nasal cromoglycate acid and salts, systemic antihistamines, glucocorticoids and their combinations, leukotriene receptor antagonists. Drugs not foreseen by international Guidelines for treating allergic rhino-conjunctivitis are not included (e.g. anti-IgE). The total number of “score points” (SP) for symptoms on one day is 21 (i.e. each of the 7 symptoms scored with a maximum of 3). The maximum SP that can be achieved by intake of medication is also set to 21 SP, subdivided into the two sub-scores for nose (max. 12 SP) and eyes (max. 9 SP). Each drug is scored considering pharmacological action (according to the corresponding ATC code), expected impact on symptoms, route of administration, the dose taken and duration of effect. Each medication score is balanced for the respective weight on symptoms and within the maximum score of each organ system. Thus scoring of medication cannot yield a higher value than symptoms at the respective organ. An example for the scoring of medications is given in Table 2. In this example a patient had a combined intake of glucocorticoid-containing nasal spray, antihistaminic eye drops and nasal spray as well as systemic antihistamine. The most potent drugs are scored first; in this case the local glucocorticoids are scored. In case the maximum score of the corresponding subscore is not reached, the score for topical or systemic antihistamines is added until the maximum score points are reached (nose: 12 SP eyes: 9 SP). In case both systemic antihistamines and local antihistamines are given, the systemic antihistamine e.g. loratadine is scored first. The sum of SP of topical, systemic antihistamines and combination-drugs containing antihistamine cannot exceed 7 SP for the subscore nose and 5 SP for the subscore eyes, respectively.

**Table 2:** Example for the calculation of the medication score (combination of topical and systemic drugs)

**Medication**	**No. of admin.**	SP* per admin.	Nose SP*	Eyes SP*	Total SP*
Levocabastine ED*	2	1	0	2	2
Levocabastine NS*	2	1	0**	0	0
Mometasone NS*	2	3	6	0	6
Loratadine	2	6	6***	5	11
Sum			**12**	**7**	**19**

* *SP* = Score points; *ED* = Eye Drops; *NS* = Nasal Spray.

** It would be 2 SP for use of Levocabastine NS alone, but in combination with the scores for Mometasone and Loratadine the maximum of 12 SP for nose is reached.

*** It would be 7 SP for use of Loratadine alone, but in combination with the Mometasone score the maximum of 12 SP for nose is reached.

#### Symptom-medication-score

The RC-ACS^©^ is obtained by adding the daily medication score to the daily symptom score leading to a range of 0 to 42 SP. Similarly, the N-ACS^©^ as well as the E-ACS^©^ is calculated. The daily E-ACS^©^ and N-ACS^©^ range from 0 to 18 SP and 0 to 24 SP, respectively.

### Pollen counts

Pollen counts (grasses, rye and mugwort) were derived from the European pollen information database (European Aeroallergen Network, Vienna, Austria) between 21 June and 17 August 2008 for the pollen traps in Lübeck and Reinbek. Pollen exposition was assessed using the 4-point scale (“None”, “Weak”, “Moderate”, “Strong”) according to the definition of the German Meteorological Service) for each week of assessment (Table 3).

**Table 3:** Assessment of pollen counts according to the definition of the German Meteorological Service

**Pollen**	**No exposure**	**Weak exposure**	**Moderate exposure**	**Strong exposure**
		**[grains/m**^**3**^**]**	**[grains/m**^**3**^**]**	**[grains/m**^**3**^**]**
Hazel	0	1-10	11-100	> 100
Alder	0	1-10	11-100	> 100
Birch	0	1-10	11-50	> 50
Grasses	0	1-5	6-30	> 30
Rye	0	1-2	3-6	> 6
Mugwort	0	1-2	3-6	> 6
Ragweed	0	1-5	6-10	> 10

### Statistics

#### Descriptive analysis

Background and demographic characteristics of subjects are summarized for both groups. Continuous variables are displayed by sample size, mean, median, standard deviation and range. Discrete variables are shown with frequencies and percentages. Missing SMS values were replaced by linear interpolation if at most 25% of the values were missing. Regarding all other parameters, the last-observation-carried-forward (LOCF) method was applied. Data management and statistical analysis were performed using the statistical analysis program SPSS Version 15.0 (SPSS Inc., Chicago, USA).

#### Validation of the RC-ACS^©^

The dataset was analyzed to measure the convergent reliability, discrimination capacity, and retest reliability, as follows.

##### Convergent reliability

Convergent reliability is the degree to which the scores of an instrument show a relationship to scores of similar instruments. The convergent reliability of the RC-ACS^©^ and the total N-ACS^©^ as well as the total E-ACS^©^ was tested by correlating the average SMS value of week 1 with the following four parameters: 1) Global Assessment of Severity of Allergy (Rating Scale 1–10); 2) Quality of Life (RQLQ); 3) number of medical consultations due to the allergy within the last 12 months; 4) the number of non-productive days due to allergic rhino-conjunctivitis and/or asthma within the last 12 months. Spearman’s rank correlations were calculated for each of the criteria 1 to 4. A significant positive correlation (p < 0.05, two-tailed) was considered as evidence for convergent validity. This corresponds in this study to a medium effect size (r ≥ 0.30) which can be regarded as a considerable correlation [10].

##### Discrimination capacity

The discrimination capacity is the degree to which the scores of an instrument can discriminate between different patient groups.

Discrimination capacity of the RC-ACS^©^ and the total N-ACS^©^ as well as the total E-ACS^©^ was assessed by comparing the average SMS values of the allergic patients (first week) and the control group, respectively. Discrimination capacity was assumed to be good if the SMS value in the allergy group was significantly higher (p < 0.05, two-tailed) than the SMS value of the control group. Significance testing was performed with the Wilcoxon Rank Sum test. Sensitivity and specificity were analyzed by a ROC-curve.

##### Retest reliability

Retest Reliability is the extent to which scores for patients who have not changed are the same for repeated measurement over time. The retest reliability was determined for the patient group only, by correlating the SMS values of the first week with the values of the second week. Comparisons were conducted using Spearman’s rank correlations.

## Results

### Socio-demographic and clinical data

A total of 122 adults (82 allergic patients and 40 healthy controls) were screened for inclusion into the study. Of the 82 patients, 81 fulfilled the in/exclusion criteria and were included in the study and 80 completed the entire study. All 40 control subjects completed the study. The socio-demographic data of both groups are shown in Table 4 and the data on medical history of allergic diseases are given in Table 5 for the patient group.

**Table 4:** Socio-demographic data of the patient and control group

	**Patient group**				**Control group**			
	**(n = 81)**				**(n = 40)**			
	**M ± SD**	**Min**	**Max**	**Median**	**M ± SD**	**Min**	**Max**	**Median**
Age (years)	30.4 ± 9.7	19.0	65.0	28.0	35.5 ± 9.1	19.0	58.0	34.0
Height (cm)	172.3 ± 8.6	154.0	195.0	172.0	173.1 ± 9.6	156.0	191.0	173.5
Weight (kg)	68.0 ± 14.7	48.0	147.5	65.0	74.8 ± 18.9	45.0	130.0	71.0
**Sex**	**n**	**%**			**n**	**%**		
Male	20	25.0			18	45.0		
Female	61	75.0			22	55.0		
**Race**	**n**	**%**			**n**	**%**		
Caucasian	77	95.1			37	92.5		
Hispanic	1	1.2			0	0.0		
African	0	0.0			1	2.5		
Asian	2	2.5			2	5.0		

*n*: Sample size of subgroup *M*: Arithmetic mean. *SD*: Standard deviation. *Min*: Minimum. *Max*: Maximum.

**Table 5:** Medical history data of the patient group

				
**Type I Allergy to**	**n**		**%**	
Grass pollen	67		83	
Dust mites	57		73	
Rye	53		65	
Mugwort	26		33	
**Duration of Allergy (years)**	**M ± SD**	**Min**	**Max**	**Median**
Any allergy disorder	12.8 ± 8.7	1.0	50.0	10.0
Grass pollen	13.7 ± 9.2	1.0	50.0	11.0
Rye	15.1 ± 9.7	3.0	50.0	12.0
Mugwort	15.0 ± 8.6	3.0	37.0	14.0
Dust mites	11.8 ± 7.2	1.0	28.0	10.0
**Allergic Illnesses**	**n**	**%**		
Allergic rhinitis	80	98.8		
Allergic conjunctivitis	69	85.2		
Allergic asthma	31	38.3		
Atopic dermatitis	9	11.1		

*n*: Number of subjects. *M*: Arithmetic mean. *SD*: Standard deviation. *Min*: Minimum. *Max*: Maximum.

### Severity of symptoms in allergic patients vs. healthy controls

The severity of symptoms in allergic patients vs. healthy controls is shown in Table 6. Clearly, patients had higher values than healthy controls. Controls had values close to the lowest possible values. Since healthy controls were non-allergic based on their medical history, symptom rating of the control patients is due to other factors than allergic symptoms.

**Table 6:** Severity of allergy in allergic patients vs. healthy controls

	**Allergic patients**			**Healthy controls**		
**Criterion**	**n**	**Mean ± SD**	**Median [range]**	**n**	**Mean ± SD**	**Median [range]**
RC-ACS^©^ (units)	80	4.3 ± 3.0	3.7 [0.0; 14.1]	40	0.2 ± 0.5	0.0 [0.0; 2.9]
E-ACS^©^ (units)	80	1.2 ± 1.3	0.9 [0.0; 5.1]	40	0.0 ± 0.2	0.0 [0.0; 1.0]
N-ACS^©^ (units)	80	3.0 ± 2.1	3.0 [0.0; 9.0]	40	0.1 ± 0.3	0.0 [0.0; 1.9]
Global Assessment of Severity of Allergy (Rating Scale 1-10)	80	3.6 ± 1.6	3.6 [1.0; 7.1]	40	1.1 ± 0.2	1.0 [1.0; 2.3]
Quality of Life (RQLQ total score)	79	1.9 ± 1.1	1.8 [0.2; 4.1]	-	n.a.	n.a.
Medical Consultations due to allergy in the last 12 months	78	1.7 ± 2.5	1.0 [0.0; 12.0]	-	n.a.	n.a.
Non-productive days due to allergy in the last 12 months	80	0.6 ± 2.5	0.0 [0.0; 15.0]	-	n.a.	n.a.

*n.a*. = not applicable.

### Discrimination capacity of the RC-ACS^©^

**Figure 1:**
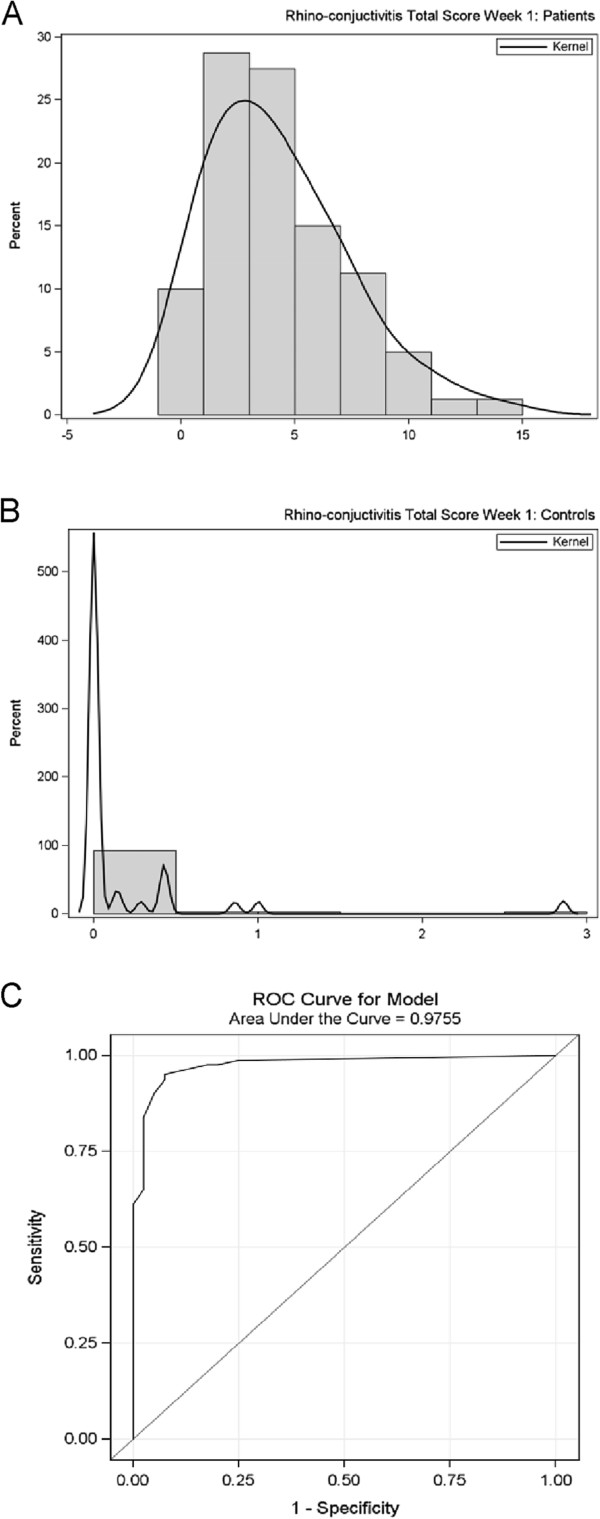
**Discrimination capacity of the SMS. **Frequency distribution of the SMS among 81 allergic patients (**A**) and 40 healthy control subjects (**B**). Panel (**C**) shows a ROC curve of the discrimination power of patients vs. controls; the area under the curve is 0.9755; the best discrimination point is 0.786, corresponding to a sensitivity of 93.8% and a specificity of 92.5%.

The patient group showed a significantly higher mean RC-ACS^©^ in comparison to the control group (Median: 3.7 range: 0; 14.1 vs. Median: 0 range: 0; 2.9; p < 0.001, two-sided Wilcoxon Rank Sum test) (Figure 1A and B). The same was seen also for the E-ACS^©^ (patient group: Median: 0.9; range: 0; 5.1 vs. control group: Median: 0; range: 0; 1.0; p < 0.001, two-sided Wilcoxon Rank Sum test) and the N-ACS^©^ (patient group: Median: 3.0; range: 0; 9.0 vs. control group: Median: 0; range: 0; 1.9; p < 0.001, two-sided Wilcoxon Rank Sum test). Thus, RC-ACS^©^, E-ACS^©^ and N-ACS^©^ separated excellently between patient and control group. The best possible SMS-cut-off-value with a sensitivity of 93.8% and a specificity of 92.5% was 0.786 (Figure 1C). In other words, 93.8% of the allergic patients had an RC-ACS^©^ of 0.786 or higher and thus could be assigned correctly to the allergy group. The same analysis has been repeated separately for E-ACS^©^ and N -ACS^©^ and provided similar results (data not shown).

### Convergent reliability of the RC-ACS©

A statistically significant (*p* < 0.0001, two-tailed) positive correlation was observed between the RC-ACS^©^ and the Global Assessment of the Severity of Allergy, the Quality of Life, and the number of medical consultations due to the allergic disease, but not with the number of non-productive days due to allergic rhino-conjunctivitis and/or asthma within the last 12 months (Table 7). However, only seven patients (8.75%) had lost at least one productive day due to allergy.

**Table 7:** **Correlation of the RC-ACS**^**©**^**, E-ACS**^**© **^**and N-ACS**^**© **^**with further assessment tools for the severity of allergy**

	**RC-ACS**^**©**^		**E-ACS**^**©**^		**N-ACS**^**©**^	
**Criterion**	**r**	**p**	**r**	**p**	**r**	**p**
Global Assessment of Severity of Allergy (*Rating Scale 1–10*)	0.6910	<0.0001	0.4873	<0.0001	0.6867	<0.0001
Quality of Life (*RQLQ total score*)	0.7573	<0.0001	0.6547	<0.0001	0.7043	<0.0001
Medical Consultations due to allergy in the last 12 months	0.3288	0.0019	0.2613	0.0192	0.2939	0.0008
Non-productive days due to allergy in the last 12 months	0.0253	0.8239	−0.0773	0.4954	0.0681	0.5482

### Re-test reproducibility of RC-ACS^©^ over time

In the patient group, the average RC-ACS^©^ values of week 1 and week 2 were almost identical (week 1 = 4.3 ± 3.0; week 2 = 4.0 ± 3.1 (Table 8)). The RC-ACS^©^ values of the first and second week correlated significantly (r = 0.8134, p < 0.0001, two-tailed). The reproducibility of the E-ACS^©^ and N-ACS^©^ (Table 8) was comparable (E-ACS^©^, week 1: 1.2 ± 1.3 vs. week 2: 1.2 ± 1.3; r = 0.7716, p < 0.0001; N-ACS^©^, week 1: 3.0 ± 2.1 vs. week 2: 2.9 ± 2.2; r = 0.7990, p < 0.0001).

**Table 8:** **Re-test reliability of RC-ACS**^**©**^**, E-ACS**^**© **^**and N-ACS**^**©**^

	**Week 1**	**Week 2**		
**Criterion**	**Mean ± SD**	**Mean ± SD**	**r**	**p**
RC-ACS^©^ (*units*)	4.3 ± 3.0	4.0 ± 3.1	0.8134	<0.0001
E-ACS^©^(*units*)	1.2 ± 1.3	1.2 ± 1.3	0.7716	<0.0001
N-ACS^©^(*units*)	3.0 ± 2.1	2.9 ± 2.2	0.7990	<0.0001

### Pollen counts

**Figure 2:**
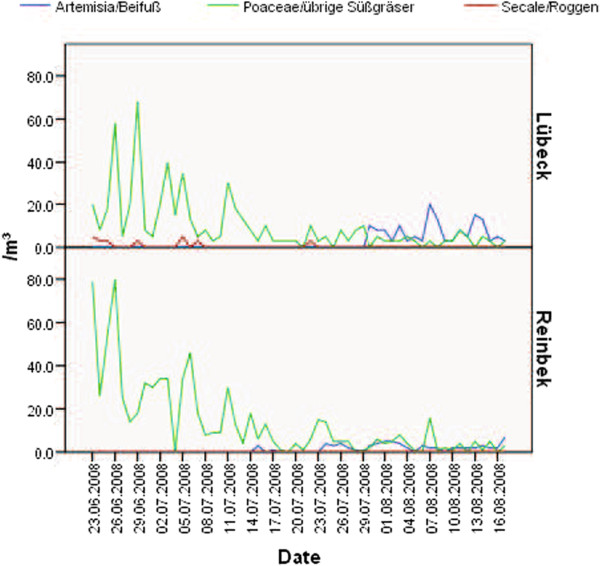
**Pollen exposure during the study period. **Pollen counts in two sites representative for this region during the whole study period.

The pollen counts of *Poaceae* in the two aerobiology stations were monitored during an eight-week period (Figure 2). Patients recorded their symptoms in a diary during this period.

## Discussion

### Major findings

This study evaluated the validity of the RC-ACS^©^, a symptom-medication score which assesses severity of nasal and ocular allergy by considering symptoms and intake of anti-allergic medication. RC-ACS^©^ is reliable, reproducible, and feasible because: 1) the convergent reliability analysis showed highly significant correlations with Global Assessment of Allergy Severity, Quality of Life, and the number of medical consultations due to allergy within the past year; 2) it discriminated significantly between patient and control groups (p < 0.001); 3) it showed good retest reliability; 4) it had an excellent sensitivity (93.8%) and specificity of (92.5%) in discriminating between patients and healthy controls. Thus, the RC-ACS^©^ is considered as a reliable and valid instrument evaluating severity of symptoms of nasal and ocular allergies. The same holds also true for the elements of the RC-ACS^©^ i.e. the N-ACS^©^ and the E-ACS^©^*.*

### Peculiarities of the RC-ACS^©^

RC*-*ACS^©^ is based on scoring nose and ocular symptoms and use of symptomatic medications. It includes a full list of relevant drugs, i.e. without any limitation in drugs. Each score can be used separately or combined. Thus, RC-ACS^©^ can be used in daily practice and real-life situations. Of note is the fact that, as with the ACS^©^, the RC-ACS^©^ balances impact of symptoms and drugs. Although with this RC-ACS^©^ a recommendation of the EMA guideline [3] can be met, it has to be considered that rhino-conjunctivitis can precede asthma [11] and often patients have both rhino-conjunctivitis and asthma [12]. Thus it is important to collect also lung symptoms in order to make assessments on development of asthma.

### The combination of symptom and medication score

The RC-ACS^©^ and the subscores E-ACS^©^ and N-ACS^©^ combine scoring of symptoms and medication by summing up both. For the symptom score and medication score separately, the retest reliability and discrimination power were excellent (data not shown). In contrast to the combined use of “Average Rhinoconjunctivitis Total Symptom Score” (ARTSS) and “Average Rescue Medication Score (ARMS)” [13] which has been specifically designed considering WAO recommendations [2], the RC-ACS^©^ weights rescue medication, and balances rescue medication and symptoms. The advantage of the RC-ACS^©^ is the individual scoring of each medication according to their ACT class, based on efficacy, mode of action, mode of administration, and duration of action.

### Other instruments

A visual analog scale as used by Bousquet and colleagues [7] seems to be an easy to use instrument to assess efficacy of e.g. specific immunotherapy. However, nowadays a visual analog scale as used by Bousquet and colleagues [7] will no longer be accepted by health authorities to achieve approval for new medications especially if used for specific immunotherapy because the EMA guideline [3] states that “…the primary endpoint has to reflect both, symptom severity as well as the intake of rescue medication”. The present article does not claim to have a comprehensive discussion of all available instruments. For this purpose the authors would like to refer to a recently published review on such instruments by Pfaar and colleagues [14].

### Cross-cultural validation

*RC-*ACS^©^ is available in German, English, Polish and other languages. Translation to other languages may be possible. The Medication Score can be used worldwide because use of ATC codes guarantees that even country-specific therapies can be coded.

## Conclusions

This study shows that the RC-ACS^©^ is a valid and reliable diagnostic tool, for assessing, and monitoring allergy severity. It considers both, symptoms and allergy medication. The structure is robust enough for using it in clinical trials and daily clinical practice. Therefore, with this validated RC-ACS^©^ there is a tool available which focuses only on rhino conjunctivitis. However, it has to be noted that the full picture of an allergic patient cannot be assessed without collecting data on lung function. With this paper we want to take the opportunity for opening a discussion on validation of such instruments and how it may be performed in the future and how it can be improved.

## Abbreviations

ACS^©^: Allergy-control-SCORE^©^; ATC: Anatomical therapeutic chemical; ARTSS: Average rhino conjunctivitis total symptom score; ARMS: Average rescue medication score; RC-ACS^©^: Rhino-conjunctivitis allergy-control-score^©^; E-ACS^©^: Eye-allergy-control-score^©^; N-ACS^©^: Nose-allergy-control-score^©^; EMA: European medicines agency; RQLQ^©^: Rhinoconjunctivitis quality of life questionnaire^©^; ROC: Receiver operating characteristics; SP: Score points; SMS: Symptom medication score.

## Competing interests

Dietrich Häfner, Hanns Meyer, Jens Kettner, and Annemie Narkus are employees of Allergopharma J. Ganzer KG, which develops and sells allergen immunotherapy products.

Kristian Reich, Ina Zschocke, and Annett Lotzin acted as a paid consultant to develop and validate a questionnaire and have received funding for research carried out in this work.

## Author’s contributions

DH participated in the design of the study and drafted the manuscript; KR participated in the design of the study and acted as investigator; IZ participated in the design and the analyses of the study; AL performed the statistical analyses; HM participated in the design of the study and checked the statistical analyses; JK participated in the design of the study and contributed to the elaboration of scoring; AN conceived the scoring system and participated in the design of the study. All authors read and approved the final manuscript.
